# Prostate cancer immunotherapy-based strategies: an updated review emphasizing immune checkpoint inhibitors

**DOI:** 10.3389/fimmu.2025.1583363

**Published:** 2025-06-18

**Authors:** Hua Lu, Zhaojun Teng, Jiajia Wang, Wenchao Zhang

**Affiliations:** ^1^ Department of Urology, Qilu Hospital of Shandong University Dezhou Hospital, Dezhou, China; ^2^ Department of Oncology, Qilu Hospital of Shandong University Dezhou Hospital, Dezhou, China

**Keywords:** prostate cancer, immunotherapy, immune checkpoint inhibitors, treatment, combined therapy

## Abstract

Prostate cancer (PC) is one of the most common cancers that is diagnosed in about 10-15% of men in old age. It seems that the current treatments are not effective, and this leads to prostate cancer becoming the second-deadliest cancer. Treatments such as chemotherapy, radiotherapy, androgen deprivation therapy (ADT), and surgery are among these treatments. However, the possibility of disease recurrence after these treatments is high. Therefore, other methods have become necessary, and PC treatment is changing. One of the methods that has received much attention today is immunotherapy. Immunotherapy includes all interventions that help to treat cancer or any other disease by affecting the immune system’s responses. For this purpose, cytokines, cell therapy, and antibody-based methods can be used. Antibody-based treatments include immune checkpoint inhibitors (ICIs), and due to the high expression of immune checkpoint (ICP) molecules on the surface of prostate cancer cells and cancer stromal cells, these treatments have yielded promising results. Also, combining them with chemotherapy, surgery, and radiotherapy can help increase their efficiency. This review first updates standard treatments’ therapeutic efficacy and risk factors. Then, we will talk about different types of immunotherapies, emphasizing ICIs.

## 1 Introduction

Metastatic prostate cancer (PC) is a leading cause of morbidity and mortality (1). Androgen deprivation therapy (ADT) remains the cornerstone of treatment for non-metastatic prostate cancer (2). However, most of these patients will progress to CRPC, which is very difficult to treat (3). There is, however, an intermediate stage where, following ADT, patients have cancer progression without detectable metastasis, termed non-metastatic castration-resistant prostate cancer (nmCRPC) (4). Awareness of nmCRPC is increasing because of ADT’s increased utilization and failure. Men with nmCRPC have a high risk of progressing to metastatic castration-resistant prostate cancer (mCRPC) and thus have limited treatment options (5). However, some treatments have recently been found beneficial, including three nonsteroidal antiandrogen agents under phase III trials (6). These agents are now FDA-approved, offering effective options alongside ADT for nmCRPC patients. The treatment of nmCRPC has improved significantly in the last ten years, with three new nonsteroidal antiandrogen agents added to ADT (7, 8). Trials like ARAMIS, PROSPER, and SPARTAN showed better metastasis-free survival (MFS) for high-risk patients (9). Continuous ADT can lead to problems for patients, such as sexual dysfunction, low mood, acute renal injury, cardiovascular disease, and increased health costs (10, 11). Intermittent ADT allows recovery of testosterone levels and may reduce these issues (12, 13). Studies show no significant differences in cancer outcomes between intermittent and continuous ADT, but intermittent therapy usually has better sexual outcomes, less morbidity, and lower costs (14). Despite this, the best way to administer ADT is still unclear, and careful patient selection is essential for achieving benefits. Other types of PC treatments have also been used, such as chemotherapy, radiotherapy, and vaccines (**Table 1**). Various vaccines have been designed to treat PC; however, only dendritic cell (DCs) vaccines have significantly progressed, and only one has received FDA approval (**Table 2**).

**Table 1:** Treatment options for prostate cancer treatment.

Treatment option	Example	Outcome	Side effects	Ref.
Chemotherapy	Docetaxel	↑ Median overall survival	1. Diarrhoea 2. Neuropathy 3. neutropenia	(234)
	Cabazitaxel	1. ↑ Overall survival 2. Improved PSA response 3. ↓ Pain 4. ↑ Progression-free survival (PFS)	1. Haematologic toxicities 2. Neutropenia 3. Nausea 4. Fatigue 5. Diarrhea	(235)
Radiotherapy (RT)	External beam RT + ADT	1. ↑ Median overall survival	1. Fatigue 2. Diarrhea 3. Nausea and Vomiting 4. Rectal bleeding 5. Skin reaction	(236)
Androgen deprivation therapy (ADT)	Enzalutamide	1. ↑ Overall survival 2. Improved PSA response 3. Improved radiographic PFS	1. Neurotoxicity 2. Arterial hypertension 3. Asthenia 4. Hot flushes	(237)
	Abiraterone	1. ↓ PSA concentration 2. ↑ Progression-free survival	1. Arterial hypertension 2. Hypokalaemia 3. Peripheral oedema	(238)
DNA repair mediators	Polymerase inhibitors (Pis) such as olaparib	1. Improved radiographic PFS 2. ↑ Objective response rate (ORR) 3. ↑ Median overall survival	1. Reversible anaemia 2. ↑ Acute myeloid leukaemia risk 3. Myelodysplasia	(239)
	Combining Pis and ADT	1. Improved radiographic PFS 2. ↑ Median overall survival	Anaemia	(240)
PTEN/AKT modulation	Capivasertib	1. ↓ PFS and PSA	May affect non-cancerous cells and lead to toxicities at therapeutic doses	(241)
	Ipatasertib	↑ Clinical outcome	NA	(242)

↓ decrease ↑ increase.

**Table 2:** Example of vaccine application for prostate cancer in phase 3 clinical trials.

Study name	Intervention Model	Estimated Enrollment	Drugs	Sponsor	Date	Status	NTC number	Key Findings
GVAX® Vaccine for Prostate Cancer vs Docetaxel & Prednisone in Patients With Metastatic Hormone-Refractory Prostate Cancer	Parallel Assignment	626	1. Chemotherapy (Taxotere and prednisone) 2. Immunotherapy with allogeneic prostate vaccine	Cell Genesys	2008	Terminated	NCT00089856	Futility analysis showing <30% chance of meeting the primary endpoint.
Vaccine Therapy in Treating Patients With Metastatic Prostate Cancer That Has Not Responded to Hormone Therapy	N/A	127	Sipuleucel-T	Dendreon	2010	Completed	NCT00005947	1. Modest toxicity profile 2. Survival benefit 3. 33% reduction in the risk of death
Docetaxel in Combination With GVAX ® Immunotherapy Versus Docetaxel and Prednisone in Prostate Cancer Patients	Parallel Assignment	408	1. allogeneic GM-CSF secreting cellular vaccine 2. Chemotherapy (docetaxel and prednisone)	Cell Genesys	2008	Terminated	NCT00133224	Accrual and treatment with CG1940/CG8711 stopped due to IDMC recommendation
Phase 3 Study of ProstAtak® Immunotherapy With Standard Radiation Therapy for Localized Prostate Cancer (PrTK03)	Parallel Assignment	711	Aglatimagene besadenovec + valacyclovir	Candel Therapeutics, Inc.	2024	Active, not recruiting	NCT01436968	Study results have not been submitted
Provenge® (Sipuleucel-T) Active Cellular Immunotherapy Treatment of Metastatic Prostate Cancer After Failing Hormone Therapy	Parallel Assignment	512	Sipuleucel-T	Dendreon	2010	Completed	NCT00065442	1. Prolonged overall survival 2. 22% reduction in the risk of death
A Randomized, Double-blind, Phase 3 Efficacy Trial of PROSTVAC-V/​F +/​- GM-CSF in Men With Asymptomatic or Minimally Symptomatic Metastatic Castrate-Resistant Prostate Cancer (Prospect)	Parallel Assignment	1297	1. PROSTVAC-V 2. PROSTVAC-F 3. GM-CSF	Bavarian Nordic	2019	Completed	NCT01322490	1. PROSTVAC was safe and well-tolerated 2. No effect on overall survival 3. No effect on alive without events
Phase III Study of DCVAC/​PCa Added to Standard Chemotherapy for Men With Metastatic Castration Resistant Prostate Cancer (VIABLE)	Parallel Assignment	1182	DCVAC/PCa	SOTIO a.s.	2021	Completed	NCT02111577	There was no difference in overall survival between the DCVAC/PCa and placebo groups

Over the past decade, the standard of treatment has been immunotherapy, whereby various forms of immune response are employed to destroy the malignant cells (15). It has had favorable results in those suffering from aggressive forms of PC (like mCRPC), with some patients achieving permanent remission (16). Other therapies, including sipuleucel-T (17) and immune checkpoint inhibitors (ICIs) (18), have also emerged as alternatives to conventional ADT and chemotherapy for the management of CRPC.

Adoptive Cell Therapy (ACT) is also moderately successful in treating different cancers (19). It utilizes specially modified T-lymphocytes to target specific tumors effectively. Modifying patient T-lymphocytes with particular antigen receptors can generate an anticancer immune reaction against PC antigens (20). Chimeric antigen receptors (CAR) support the preparation of artificial T-cell receptors for ACT in PC patients (21).

However, immunotherapy tends to be less effective against prostate cancer than against other malignancies, such as non-small-cell lung cancer and melanoma, due to the suppressive nature of the tumor environment and lower T-cell content (22–24). Nevertheless, certain subsets of prostate cancer patients with specific characteristics have indeed responded well to ICIs (16). However, in many studies, ICIs have been used in combination with other therapies, such as chemotherapy, radiotherapy, and vaccines (25, 26). In this review, we first discuss the types of immunotherapies for prostate cancer and then explain them by classifying immunotherapy into two categories: cell therapy and antibody therapy. Then, we will discuss the limitations, challenges, and suggestions, and conclude.

## 2 Immunotherapy strategies

**Figure 1:**
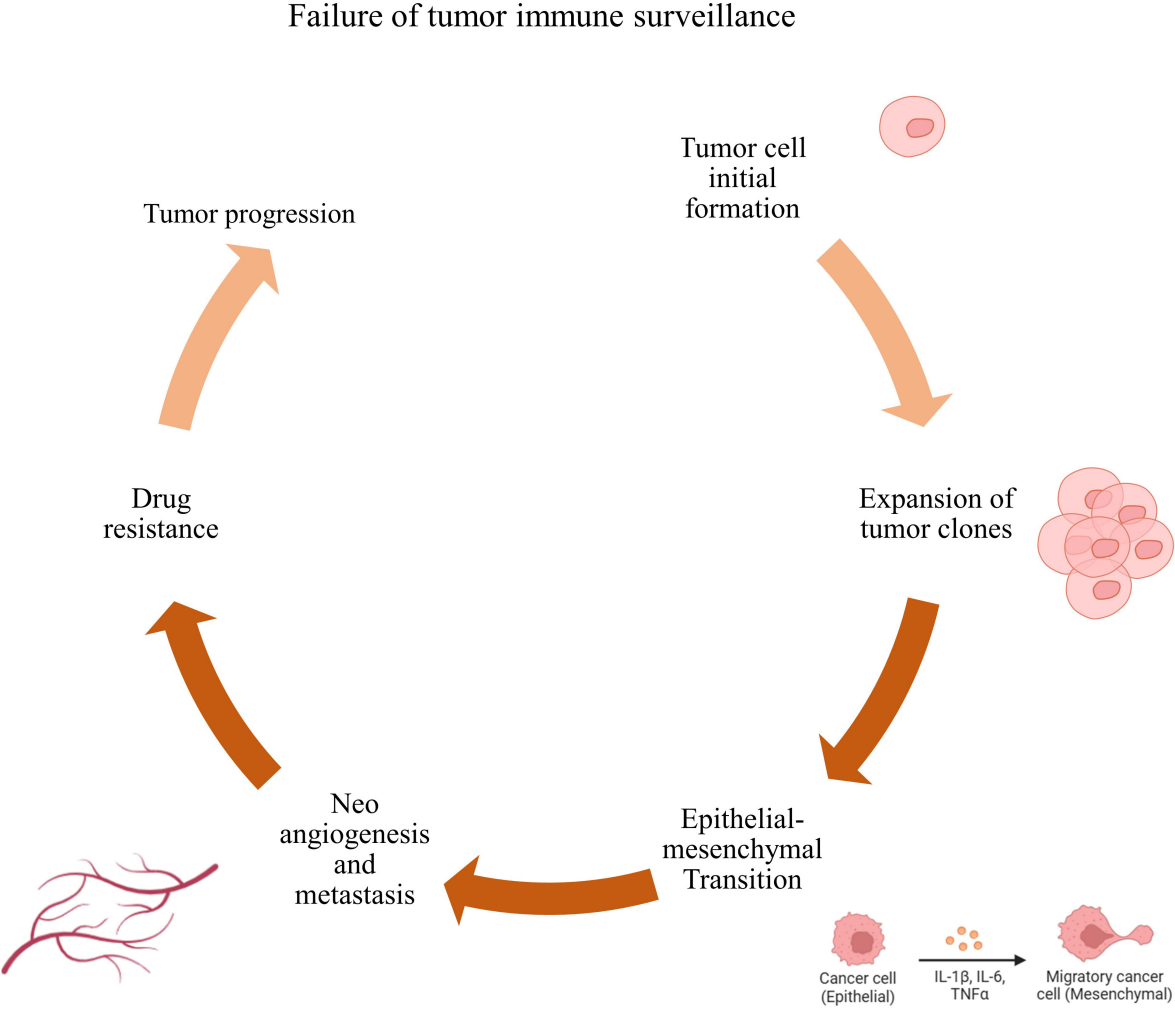
Failure of immune surveillance and tumor progression. Failure to kill tumor cells by immune cells leads to their rapid proliferation, increased invasion, metastasis, and resistance to tumor cell treatment, which in turn leads to tumor progression.

Immune surveillance failure contributes to tumor development (27) (**Figure 1**). Tumor cells can escape T-cell responses by several mechanisms. Active immunotherapy tries to augment the immune response against cancer (28). Prostate cancer is unique in that it is one of the tumors recognized by the immune system, as evidenced by tumor-infiltrating lymphocytes, and therefore is a candidate for immunotherapy (29). PC has defined antigens that enable targeting without inducing widespread autoimmune responses. There are several approaches to immunotherapy, and only Sipuleucel-T is an FDA-approved modality (17). Immunotherapy includes all interventions that increase the immune system’s potential to respond to cancer cells (30, 31). These types of interventions are categorized based on the type of intervention and the part of the affected immune system. In general, these types of treatments are divided into two categories: cell therapy and antibody therapy.

### 2.1 Cell therapy

In cell therapy for PC, different therapeutic strategies and cells are used. Among the most critical cells used in treating PC are DCs (32), tumor-infiltrating T cells (TILs) (33), and chimeric antigen receptor-expressing T cells (CAR-T) (34). Although the therapeutic uses of CAR-T cells seem to be confirmed for the treatment of blood cancers, their therapeutic efficacy for solid tumors is still under investigation.

#### 2.1.1 Dendritic cell -based treatments

Vaccines have played a crucial role in eradicating various infectious diseases and saving millions of lives. These vaccines work through the induction of specific immune responses against attenuated or killed germs to protect against actual infections (35). So far, developing effective anti-tumor vaccines has been challenging and requires further improvement (36).

The DCs are one of the potent immune cells contributing to cancer immunotherapy by inducing specific immune responses (37). Various approaches have been tried to use DCs as therapeutic vaccines, employing either whole tumor cells or specific protein fragments.

Currently, sipuleucel-T (sip-T) represents the only FDA-approved therapy for men with asymptomatic or minimally symptomatic metastatic castrate-resistant PC (38). Its toxicity profile is generally favorable, making it more attractive than docetaxel. In April 2010, the FDA approved sip-T for the treatment of PC. However, patient access to this type of treatment is limited, and side effects such as a fast heartbeat, fever, rash, and joint pain may occur following treatment (39).

Prostate acid phosphatase (PAP) is also used to design a therapeutic vaccine that elicits an immune response against prostate cancer cells and targets (40). Another DC-based vaccine was developed to generate T cells that recognize and kill cancer cells that express PAP, mainly from malignant prostate tissue post-surgery (41). A study used a recombinant fusion protein vaccine expressing PAP with GM-CSF (sip-T) to activate autologous peripheral blood mononuclear cells (PBMC). After processing, the cells containing activated APCs (especially DCs) are infused back into the patient (42). Given the success achieved in treating PC with this type of vaccine, many researchers have sought to increase the potential of this treatment. The results of studies have shown that this type of treatment can lead to increased migration of tumor antigen-specific T cells to tumor tissue (42). In another study by RK Pachynski et al., patients were divided into two groups after receiving sip-T. One group received recombinant human IL-7 (4 injections per week for 6 weeks); the other group received a placebo (43). The results of this study proved that IL-7 injection can increase the therapeutic efficacy of sip-T. In patients receiving IL-7, the number of CD8^+^, TCD4^+^ T cells, and NK cells was significantly increased compared to the control group (43). In this study, due to limited access to recombinant human IL-7, the number of patients decreased during the trial (from 80 to 54), and no association between immune findings and clinical outcomes was reported.

Another DC-based vaccine for PC is stapuldencel or DCVAC/PCa, and is obtained from autologous DCs derived from PBMC of patients exposed to a lysate of human prostate adenocarcinoma cell line (LNCaP) (44). Various studies show that this type of treatment is highly safe and well-tolerated. They can also lead to an increase in the population of prostate-specific Antigen (PSA)-specific T cells in patients (45). Different combinations of this cell therapy have also been used during various trials. For example, in a study by Vogelzang et al., Stapuldencel was used with docetaxel to treat metastatic PC refractory to treatment. This study’s results show that this combination can increase overall survival (OS) in patients compared to a single treatment (46). Also, in another Phase I/II study, it has been shown that the use of autologous DC vaccine as an adjuvant after prostatectomy can lead to a decrease in relapse in patients (47).

Although previous DC vaccine-based therapies have succeeded, given their high potential in PC treatment, studies on their therapeutic use are ongoing, and different antigens are used to prime them (48). A study used MAGE-A2 long peptide (LP) to induce maturation in DCs. The results of this study show that the use of DCs + MAGE-A2 LP leads to activated DCs and increases their ability to activate CD8^+^ T cells. Also, T cells’ cytotoxic ability and IFN-γ production are increased (49). NY-ESO-1 as an antigen has also been used to produce DC vaccines against prostate cancer (50). For this purpose, after isolating monocytes from the bone marrow of mice and inducing their differentiation into DC, a cytokine cocktail and a fusion protein containing NY-ESO-1, secretin-penetration, and ubiquitin (SNU) were used (51). The results show that this DC taxon effectively stimulates mouse PBMCs to produce inflammatory cytokines and increases their cytotoxic ability in co-culture with tumor cells. Overall, the observed cases increase the ability of T cells to mount a specific anti-tumor response and are a suitable option for transfer to clinical studies (51).

Therefore, identifying specific and dominant antigens related to PC and using them to prime T cells can help in its treatment. It is also suggested that DCs be primed with a specific antigen cocktail for PC and used for treatment. This proposed therapy can simultaneously stimulate different clones of PC-specific T cells, leading to tumor regression. An essential point about DC-based vaccines for prostate cancer is that this treatment is often used after surgery to prevent recurrence.

#### 2.1.2 Tumor-infiltrated lymphocytes

**Figure 2:**
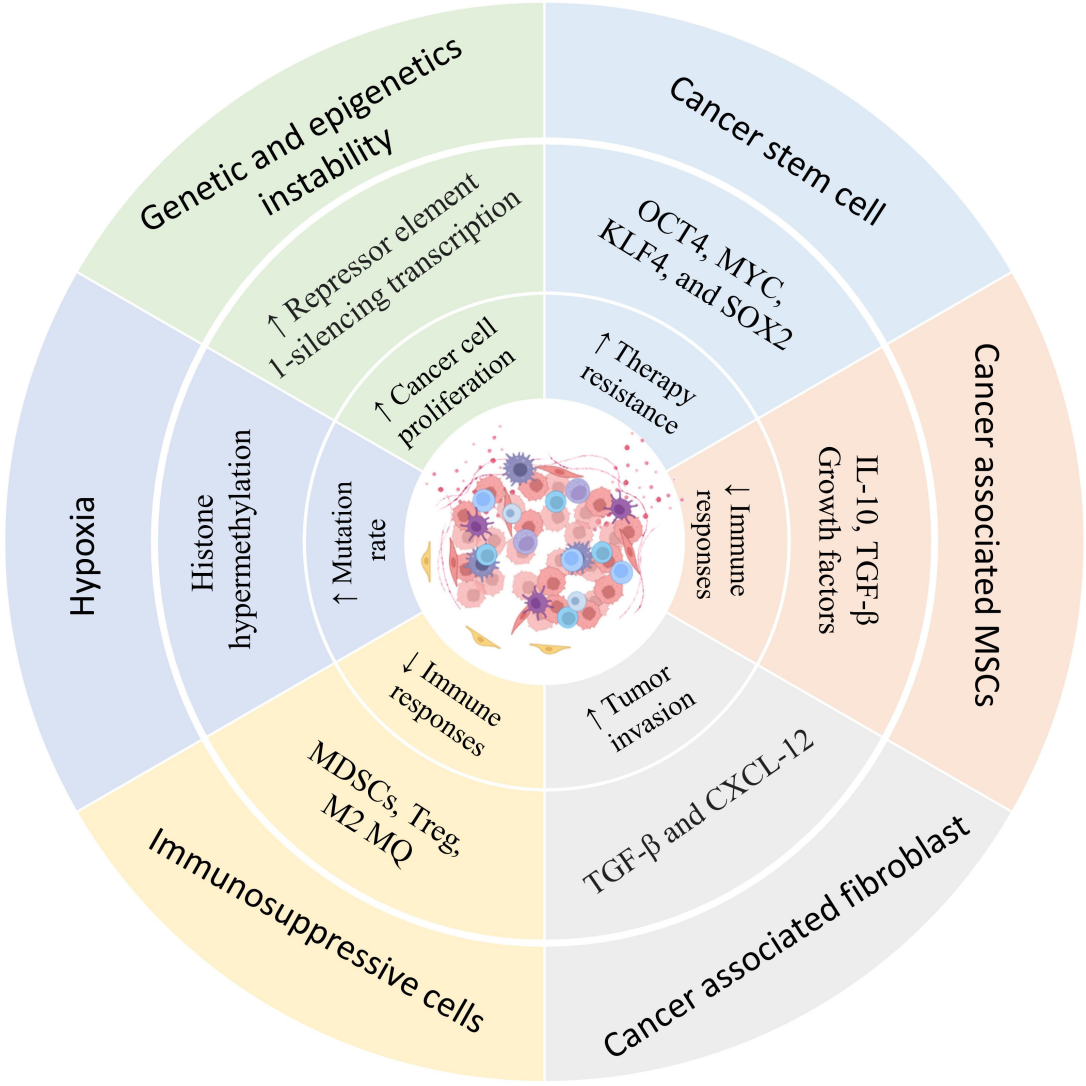
Heterogeneity of the prostate cancer microenvironment. The presence of different types of immune regulatory cells in the tumor tissue environment, hypoxia, mutations, and epigenetic changes resulting from food starvation, lead to changes in the characteristics of cells in the tumor microenvironment that contribute to tumor growth. Understanding this heterogeneity can help in tumor treatment and the selection of appropriate therapy.

Human tumors express antigens recognizable by T and B lymphocytes, and such recognition forms the basis for new immunotherapeutic approaches directed at tumor-associated antigens (TAA) (52). However, attempts to develop successful cancer vaccines have met with limited success. Although vaccination can expand tumor-reactive T lymphocytes, clinical responses have been seen in only a few patients. This is unsurprising because relatively few lymphocytes may reach the cancer, and tumors have found ways to evade the immunological attack (53, 54). Tumor cells can avoid or escape immune detection by losing antigens, producing immunosuppressive molecules, and recruiting suppressive immune cells (55, 56). Mixed responses have been frequently observed in patients receiving immune therapy, where some tumors regress while others progress. This is poorly understood, but differences in the tumor-immune cell interaction could be one factor (57). The tumor microenvironment (TME) generally does not support T lymphocyte activities, as various studies have documented that tumor-residing T lymphocytes often have impaired functionalities (58, 59) (**Figure 2**).

Another essential immune regulatory aspect in the context of tumors is amino acid metabolism (60). For instance, it has been documented that tumors induce an enzyme that degrades tryptophan, which has been associated with immune evasion mechanisms (61). Inhibiting this enzyme in certain studies has helped reverse some tumor-induced immune dysfunctions (62). L-arginine metabolism is also altered in tumors. In immune cells, L-arginine is metabolized by several enzymes that affect immune responses (63). High activity of these enzymes has been associated with a range of cancers and appears to support tumor growth, yet they can also suppress T cell responses. It is, therefore, tempting to speculate that the tumor-associated metabolism of L-arginine inhibits T cell function, and indeed, inhibitors directed against these pathways seem to reconstitute T cell functions in PC (64).

Given the immunosuppressive environment in the TME, it appears that extracting tumor-specific lymphocytes from patients and reactivating them may help treat PC in two ways. First, cells expanded outside the body and no longer received inhibitory signals induced by tumor cells, which led to increased activation (65). Also, increasing the number of these cells outside the body and re-transfusing them into patients increases the number of activated tumor-specific lymphocytes and helps in tumor regression.

However, in the case of prostate cancer, various studies have shown that isolating and activating TILs from patients is challenging. However, a study by Sharon Yunger et al. has shown that TILs can be isolated from prostate tumor tissue. These cells also expanded *in vitro* after activation, and their ability to produce IFN-γ and tumor killing (in co-culture with tumor cells) was also increased (66).

#### 2.1.3 Chimeric antigen receptor-expressing T cell

One of the techniques for empowering a patient’s T cells is to add a CAR-based receptor on their surface for specific recognition and destruction of cancer cells (67). These CAR T cells are often referred to as “living drugs” because they can grow and become long-lived memory cells (34). Unlike the regular T cells, which depend on the human leukocyte antigen (HLA) complex, CAR T cells recognize targets in an HLA-independent manner (68). When they bind to an antigen, they activate and release different substances, like cytokines, that destroy the target cells. The parts that make up CARs are numerous, including an antigen-binding domain and a linker region, which place the binding domain for better positioning in recognizing tumor cells (69). The type of linker and the length could influence the effectiveness of the CAR T cell.

Another part of a CAR is the transmembrane domain that can made of CD3, CD4, CD8, or CD28 transmembrane domains and anchor CARs in the cell membrane and contribute to sending signals for T-cell activation upon recognition of a target antigen (70). The final part is the intracellular domain of CARs, which has undergone the most variation over generations. First-generation CARs contained only the CD3 ζ-chain or FcϵRIγ as their signaling domain. These first-generation CARs demonstrated inferior T-cell activation (71, 72). Second- and third-generation CARs have been engineered by incorporating one or two co-stimulatory domains, commonly using CD28, 4-1BB, ICOS, or OX40 fragments (71). These co-stimulatory domains enhance T-cell activity and increase the efficacy of CAR T cells in clinical trials. The CD28 co-stimulatory CARs are more cytotoxic, while the 4-1BB CARs retain a more long-lasting memory function (73, 74).

To date, seven CAR T cell products have been approved by the FDA for treating lymphoid malignancies (75): YESCARTA for large B-cell lymphoma in 2017 (76), KYMRIAH for B-cell precursor acute lymphoblastic leukemia (ALL) in 2017 (77), TECARTUS for mantle cell lymphoma in 2020 (78), BREYANZI for large B-cell lymphomas in 2021 (79), ABECMA for multiple myeloma in 2021 (80), CARVYKTI for multiple myeloma in 2022 (81) and AUCATZYL for ALL in 2024 (82). Much less impressive results have so far emerged from CAR T cell therapy of solid tumors, where few complete responses have been seen in high-risk neuroblastoma or metastatic rhabdomyosarcoma. PSCA, PSMA, and EpCAM are currently target antigens for research in prostate cancer using CAR T cell approaches.

##### 2.1.3.1 Prostate-specific membrane antigen

PSMA currently represents the most popular CAR T cell therapy target in prostate cancer (21). It is a glycoprotein in prostate cells, but is also expressed in other tissues, including salivary glands and the nervous system (83). PSMA is overexpressed on the surface of prostate cancer cells and relates to the aggressiveness and progression of the disease (84). Its presence in other tumors suggests that PSMA-targeting CAR T cells could also help treat these cancers. Several PSMA-specific monoclonal antibodies have been developed to generate these CAR T cells, and 3/F11-derived versions have demonstrated better activation and killing of prostate cancer cells (21, 85). Animal model studies showed that the earlier versions of CAR T cells had low effectiveness, but newer types demonstrated moderate activity. High-dose systemic administration had some effects, while direct administration of CAR T cells into the tumors completely eradicated the tumors (86). However, these study results are still preclinical; additional research must translate them to humans.

To prevent these excessive responses, the research group led by Gaia Zuccolotto et al. modified the CAR by adding a stimulatory domain to its cytoplasmic structure, so that excessive stimulation leads to the induction of cell death in these cells and prevents harmful responses in the body. On the other hand, studies have used suppression of transforming growth factor-β (TGF-β) signaling to increase the efficacy of PSAM-specific CAR T cells in the treatment of prostate cancer (87–89). These *in vitro*, preclinical, and clinical studies collectively indicate that this strategy can improve the therapeutic efficacy of PSMA-specific CAR T cells.

Many studies have utilized the combined use of PSMA-specific CAR T cells with chemotherapy to enhance the efficacy of these cells. In various studies, docetaxel was combined with PSAM-specific CAR T cells to treat mice with PC. The results showed that using this combination therapy could improve the condition of sick mice, their survival, and reduce the side effects of chemotherapy (90, 91). Another method to increase the efficacy of CAR T cells expressing the antigen against PSMA is using duo-CAR T cells. In a study by D Wang et al., CAR T cells expressing receptors for PSMA and IL-23 were used. The results show that this treatment can increase the recruitment of TCD8^+^ and TCD4^+^ cells to the tumor site (92).

##### 2.1.3.2 Prostate stem cell antigen

Another target is PSCA, which is associated with various cancers, including prostate cancer (93). It is overexpressed in the advanced stages of the disease, and research indicates it may promote tumor growth (94). Second-generation CAR T cells targeting PSCA have been studied, showing that those with different co-stimulatory domains affect T cell performance differently (95). Intratumoral injections initially eliminated tumors, but afterward, the tumors relapsed because they lost PSCA. This implies that combinations with other therapies may improve efficacy. Overall, results suggested that too much activation of CAR T cells may harm their long-term tumor control. Another study used Vγ9Vδ2 TCR-enriched PSCA-specific CAR T cells expressing the receptor to treat PC. The γδ CAR-T cells in this study reduced prostate cancer bone metastasis (96).

##### 2.1.3.3 Epithelial cell adhesion molecule

EpCAM is a type I glycoprotein expressed on epithelial cells and plays an essential role in cell adhesion, migration, and differentiation (97, 98). EpCAM is frequently overexpressed in prostate cancer tissues compared to benign tissues and healthy controls. However, studies on its association with clinical parameters in prostate cancer show conflicting results: while some report an association with poor prognosis, others show no such correlation. In one preclinical study, using EpCAM-targeting CAR T cells resulted in effective tumor control in a mouse model (99). Using EpCAM-targeting CAR T cells generated from peripheral blood T cells of prostate cancer patients improves OS. It reduces tumor size in PC3 (low EpCAM expression) and PC3M (high EpCAM expression)-induced prostate cancer mouse models. It seems that although PC3 tumor cells express low levels of EpCAM due to their high expression in cancer stem cells, using these CAR T cells can lead to improving symptoms in PC mouse models (99). However, some studies have shown that using this type of CAR T cell can cause side effects such as weight loss and cytokine release syndrome. Studies also show that due to the high level of EpCAM expression in lung tissue, using EpCAM-targeting CAR T cells can lead to pathogenesis and damage to lung tissue (100).

##### 2.1.3.4 Natural killer group 2D ligand

*Natural killer group 2D ligand (NKG2DL) is a potential* target for CAR T cell therapy in prostate cancer. Studies in mice have shown that targeting NKG2DL with CAR T cells can control tumor growth and improve survival by over 80%. Combining CAR T cells with IL-7 enhanced effectiveness. However, high or repeated doses may be needed, which could be challenging in humans. The solid tumor microenvironment poses a significant obstacle; however, directly injecting CAR T cells into or near tumors could be a promising approach for patients. Combination strategies of this type of T cell work with IL-7 (101), IL-27 (102), and anti-PD-L1 monoclonal antibodies (103) have also shown therapeutic efficacy in *in vitro* and animal studies.

##### 2.1.3.5 Other antigens

Six-transmembrane epithelial antigen of prostate-2 (STEAP2) is a protein highly expressed by prostate tumor cells, and its use for targeting CAR T cells can be effective (104). In the study by P. Zanvit et al., the receptor that binds to STEAP2 was used as the CAR. These cells were also engineered to express a trap receptor for TGF-β and prevent the induction of fatigue by this cytokine in the produced CAR T cells. The results show that these CAR T cells can decrease tumor cell growth (105).

B7-H3 (CD267) is also expressed as an immune checkpoint by tumor cells, especially prostate cancer stem cells (106). Yida Zhang et al. have investigated its effects *in vivo* and *in vitro* by producing B7-H3 CAR T cells. The results of this study suggest that fractionated irradiation (FIR) combined with B7-H3 CAR T can inhibit tumor growth in a mouse model (107).

### 2.2 Antibody-based immunotherapies

Antibodies are one of the primary therapeutic methods inhibiting the various pathways tumors use to escape the immune system (108). After production, antibodies can be efficiently isolated and efficiently isolated and, depending on the antibody-recognized antigen, used to treat various diseases. In the case of prostate cancer, growth-inhibitory antibodies, metastasis-inhibitory antibodies, and angiogenesis-inhibitory antibodies can be mentioned (109)Among the antigens for which antibodies have been produced to treat PC is PSMA. Additionally, due to their targeted function, antibodies are now used in engineered forms. Among the antibodies engineered to increase their therapeutic efficacy are Radionuclide-conjugated antibodies, Antibody-drug conjugates, and T–cell–recruiting bispecific engagers, which we will discuss in more detail below.

#### 2.2.1 PSMA-antibody-based treatments

Antibodies J591 and J415 have been produced against PSMA, one of the main tumor-specific antigens in PC, which can bind to the extracellular domain of this antigen with high affinity (110). Therefore, they are commonly used in various applications, including those engineered for the treatment of PC (111). The first attempts to conduct studies with the 7E11 antibody (for the intercellular domain of PSMA) were unsuccessful, but the development of J591 targeting the extracellular part of PSMA was a big step forward. The studies performed with 177Lu-J591 (as β-emitting radiopharmaceuticals conjugated to J591) demonstrated effective targeting and better clinical responses (112). Trials performed with 225Ac-J591 (as α-emitting radiopharmaceuticals conjugated to J591) also demonstrated safety and preliminary signs of effectiveness (113).

The first PSMA antibody-drug conjugate (ADC) was MLN2704, using J591 to deliver drug maytansinoid 1 (DM1). Phase 1/2 studies demonstrated activity in metastatic castration-resistant prostate cancer; however, development was halted due to neurotoxicity (114). Another study used the microtubule-disrupting agent monomethyl auristatin E (MMAE) conjugated with anti-PSMA antibody and determined 2.5 mg/kg as the maximum tolerated dose (115). Several other PSMA-ADCs are in development because of the limited therapeutic window despite confirmations of safety and efficacy.

Pasotuxizumab is a bispecific T-cell engager (BiTE) immune therapy that engages T cells by binding to CD3, enabling them to attack prostate cancer cells that express PSMA. Studies have shown promise in activating T cells and delaying tumor growth. The study by Hummel et al. aims to assess the safety, tolerability, and maximum tolerated dose of pasotuxizumab as a single agent in patients with advanced PC through phase I (116). This study is the first report that shows that BiTE immune therapy is effective as a monotherapy against prostate cancer and, in fact, against any solid tumor. Early results in patients with advanced PC are encouraging. JNJ-081 is another BiTE that, like the previous study, was produced against CD3 T cells and PSMA tumor cells and has completed its phase 1 clinical trial (117).

On the other hand, the EN Glud and colleagues’ research group designed T-cell engagers that bind to CD3 on one side and PSMA on the other, with specificity and the ability to bind to FcRn. The results of this study showed that these T-cell engagers are recycled by FcRn and, based on the level of PSMA expression, have acceptable cytotoxicity on tumor cells by activating T-cells (118). Given that this type of treatment can produce large amounts of cytokines and the resulting side effects, the K Dang research group and colleagues solved this problem by lowering the binding affinity of the T-cell engager to CD3 (119).

#### 2.2.2 Antibody against other antigens

The results of various studies show that the expression of N-cadherin by prostate cancer cells can have a direct relationship with the ability to metastasize and resistance to tumor treatment (120). For this reason, in the study conducted by H Tanaka et al., an antibody produced against the extracellular domain of N-cadherin was used to treat PC. The results of this study show that the antibody against N-cadherin is effective *in vitro* and *in vivo*, and in addition, it can reduce the growth of tumor cells and reduce the ability to metastasize and resist treatment. In addition, the establishment of tumor xenografts in an animal model was also reduced. Overall, the results of this study indicated that N-cadherin is one of the main factors involved in treatment resistance in castration-resistant prostate cancer (121).

Many studies have shown that prostate tumor cells express enolase-1, thereby increasing their migratory ability. In a study conducted by ML Chen et al., an antibody against enolase-1 was used to treat PC. The use of this antibody in mice resulted in the inhibition of tumor cell growth, specifically in the PC3 cell line. Further investigation to determine the exact effect of this antibody showed that the antibody against enolase-1 could exert its antitumor action by inhibiting tube formation (mediated by VEGF-A) and preventing bone metastasis (122).

As discussed throughout this section, different antibodies directed against different antigens can inhibit tumor growth and affect tumor cell function in PC models. However, none of the studies have compared the efficacy and safety of the discussed antibodies. When comparing antibody-based therapies, factors to consider include the level of target antigen expression on the surface of tumor cells, the functional activity involved after antibody binding, the time required for production, the cost of production, and the availability after clinical trials.

## 3 Immune checkpoint inhibitor-based treatments

The development of immune checkpoint inhibitors has transformed cancer treatment by permitting long-term survival in patients with advanced disease and new options in earlier stages of the disease (123).

Essential points that relate to ICIs’ use in prostate cancer include the fact that this disease has low tumor mutational burden (TMB)and a low somatic mutation frequency compared to other highly responsive diseases of this type of treatment, like melanoma or lung cancer (124); thus, relatively fewer immune cells, including tumor-specific T cells, would migrate toward the tumor. Moreover, hypoxic zones in the tumor site cause reduced T-cell infiltration by affecting factors such as low pH and nutrient deficiency (125). These hypoxic zones further lead to increased myeloid-derived suppressor cells (MDSCs) and tumor-associated macrophages (TAMs), inhibiting the immune response (126, 127). The T cell population mainly includes immunosuppressive regulatory T cells (128). Other factors that impede anti-tumor responses are the loss of MHC class I and the frequent loss of PTEN, thereby decreasing the efficacy of immunotherapy in prostate cancer (129).

In addition to the above, studying tumor characteristics using biomarkers can be crucial and aid in selecting the appropriate type of immunotherapy. Today, many biomarkers such as hot or cold tumor, microRNAs, cell-free DNA, circulating tumor DNA (ctDNA), CD8/CD4 ratio, studying the population of CD8-positive anti-tumor specific T cells, and also exploring the altered microbiome of patients are mentioned (130, 131). Given that changes in the levels of DNA and extracellular RNA in the blood can indicate the level of malignancy, metastasis, resistance to treatment, and treatment selection, they are of great importance. It also seems that the altered microbiome (dysbiosis) can affect the initiation and spread of PC, and treatment and response to treatment through the production of various metabolites (132, 133).

Therefore, considering the immunosuppressive properties of the tumor microenvironment, the use of ICIs can compensate for the suppression induced by this environment and help treat prostate cancer.

### 3.1 Anti-programmed death-1/ligand antibody

**Figure 3:**
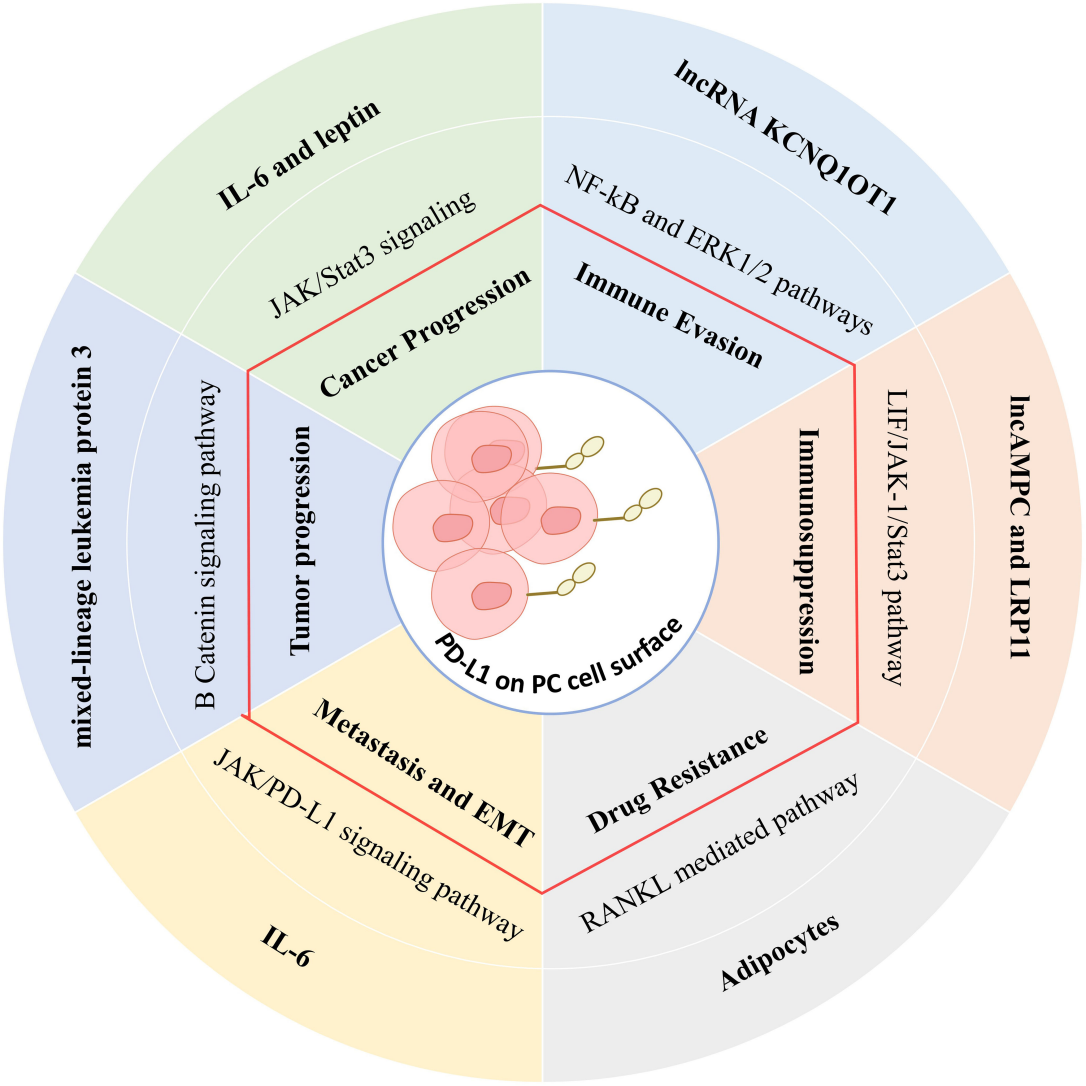
The effect of the tumor microenvironment on PDL-1 expression through various signaling pathways in tumor cells and its immunosuppressive effects.

PD-1 is expressed on activated T, B, and NK cells and has two ligands, PD-L1 and PD-L2 (134). The interaction of PD-L1 with PD-1 inhibits T cell activation and converts naive T cells into regulatory T cells that suppress excessive immune responses against normal cells during antigen-specific responses (134). Tumors exploit PD-L1 to evade T cell attack by inducing the energy or death of the effector T cells (**Figure 3**). PD-1 shares a structural similarity with the molecules CD28 and CTLA-4, also implicated in T cell activation and inhibition (135).

High levels of PD-1/PD-L1 expression are associated with significant clinical features in prostate cancer (136). The promoter of PD-1 is hypermethylated in cancer tissues compared with normal prostate tissues, establishing a negative correlation between PD-1 methylation and PD-1 mRNA expression (137). Higher PD-1 methylation is associated with higher preoperative PSA scores, higher Gleason grades, and more advanced tumor categories, serving as an adverse prognostic factor for biochemical recurrence-free survival (RFS) (138). Methylation of PD-1 is also associated with androgen receptor activity and ERG gene fusion, suggesting a regulatory role in autoimmune responses (137). The study of PD-1 promoter methylation may help identify patients who could benefit from adjuvant therapy after surgery. Essential research evidence has shown that PD-L1 is highly expressed in prostate cancer tissues compared to normal tissues, with over half of the tested cases showing moderate to high expression (139, 140). The expression of PD-L1 in aggressive cancers correlates with tumor proliferation, Gleason scores, and androgen receptor expression (141). It is also known to be an independent negative predictor of biochemical recurrence. What is more, high-risk patients with highly expressed PD-L1 have an impaired prognosis after hormonal treatment following surgery. One study showed that PD-L1 was more frequently expressed in high-stage tumors or lymph-node-positive cases, whereas PD-1 expression was independent of tumor stage (142).

High PD-L1 expression is associated with unfavorable survival and, specifically, with aggressive prostate cancer (143). Moreover, PD-L1 is also expressed on both tumor-associated nerves and circulating tumor cells, and its tumor expression correlates with clinical progression risks (144). Finally, exosomal PD-L1 has not been fully elucidated regarding predicting the response to anti-PD-1 therapy in prostate cancer (145).

The preclinical studies related to PD-1/PD-L1 expression in prostate cancer have investigated the efficiency of checkpoint inhibitors. The results have been inconsistent, at least in part because the positivity criteria for PD-L1 have varied. One such study reported the presence of PD-L1 in 92% of prostatectomy specimens but without relation to cancer outcomes (146). Other studies showed variable rates of PD-L1 expression; some of these mentioned its possible prognostic significance for biochemical recurrence (147). The results also varied widely in different patient samples and settings.

A 17.4% objective response rate was demonstrated in the single-arm, phase Ib study, KEYNOTE-028, using pembrolizumab among 23 heavily treated mCRPC patients with measurable disease and a PD-L1 expression of at least 1% (148). Three of the four who showed partial response had an extraordinary decline in their PSA levels. The KEYNOTE-199 trial studied pembrolizumab as monotherapy in three separate groups within the mCRPC population according to their PD-L1 status (149). Median OS by blinded independent central review was 9.5 months for PD-L1-positive, 7.9 months for PD-L1-negative, and 14.1 months for non-measurable disease (149). As a single agent, atezolizumab demonstrated safety with clinical activity, including 55. 6% at a 12-month OS rate with a median OS not yet reached in this group of 15 heavily treated mCRPC (149). However, it is important to note that the lack of PD-L1 enrichment may have contributed to limited responses in this study.

Studies show that human Vγ2Vδ2 (or Vγ9Vδ2) T cells can recognize specific metabolites associated with isoprenoid biosynthesis and thus help to fight infections and tumors (150, 151). Bisphosphonate treatment enhances these metabolites, enabling Vγ2Vδ2 T cells to recognize tumors independently of MHC expression or mutation load (152). Immunotherapy with these T cells has been tested in more than 400 patients in trials and is associated with limited side effects but also limited remissions. This study is focused on adding PD-1 checkpoint blockade to Vγ2Vδ2 T cells in a PC-3 prostate tumor model in mice. Results show that blocking PD-1 improves the efficacy of T cells and causes a significant reduction in tumor volume (152).

### 3.2 Anti-CTL-associated antigen-4 antibody

T-cell activation requires specific antigenic peptide recognition by T-cell receptors and costimulatory signals (153). T cells express the CD28 and CTLA-4 receptors that interact with ligands on antigen-presenting cells (APCs) (154). While CD28 engagement activates T cells, CTLA-4 engagement inhibits them. Blockade of the CTLA-4 interactions has shown promise in enhancing immune responses in preclinical models (155). Indeed, *in vivo* studies have confirmed that CTLA-4 blockade can effectively induce tumor rejection in mouse PC models. In a model of prostate cancer, the administration of an anti-CTLA-4 antibody diminished the risk of metastatic relapse after tumor resection (156). Few toxicities from CTLA-4 blockade were observed, mainly confined to prostatitis and vitiligo, with no significant findings in primate or human tissue studies (157).

In a pilot trial, James P. Allison et al. used a single-dose anti-CTLA-4 antibody (ipilimumab) in metastatic PC to test its safety and efficacy (158). The goals of this trial included assessing safety, potential autoimmune toxicity, and changes in PSA levels. Generally, in metastatic PC patients, the therapy activity is well-gauged when PSA decline is considered equal to or higher than 50% (158). During such a study, 2–14 patients had declined at a rate above 50%. The contribution of CTLA-4 blockades and steroid therapy to this PSA decline is poorly defined. Recent data indicate that CTLA-4 blockade can induce anti-PSA antibodies that clear PSA (158).

Immunological studies show that using the CTLA-4 blocker can increase the number of TCD4^+^ ICOS^hi^ cells. These cells can produce IFN-γ and contribute to activating CTLs and NK cells and their antitumor function (159). However, anti-CTLA-4 immunotherapy does not remove FOXP3^+^ Treg cells in tumors, indicating that modifying mAbs or their combinations might improve efficacy (160).

### 3.3 Anti-TIM-3 antibody

T-cell immunoglobulin domain and mucin domain-containing molecule 3 (TIM-3) is a surface marker specific for Th1 CD4^+^ T cells. TIM-3 is expressed mainly on fully differentiated Th1 lymphocytes but not Th2 cells (161). TIM-3 may not affect the differentiation of T cells, but it plays an essential role in the transport of Th1 cells. It negatively regulates Th1 and Th17 responses and is critical in T-cell exhaustion, particularly in tumors such as NSCLC (162, 163). TIM-3 levels in patients may relate to PC patients’ survival. It showed higher TIM-3 expression on CD4^+^ and CD8^+^ T cells in PC patients’ blood and tumor tissue. These levels relate to the advanced disease stage, a factor of poor prognosis (164). It seems that the overall expression of TIM-3 by TCD8^+^ cells leads to dysfunction of these cells in prostate cancer (165).

The results of the study conducted by J. Harding et al. show that the use of LY3321367 as a monoclonal antibody blocking TIM-3 functions has safe and tolerable effects on patients (166). Further studies show that this treatment can increase the CD8 expression in patient biopsies. Given that CD8 is observed more often on T cells and also on DCs, it seems that this treatment can lead to an increase in the recruitment of immune cells involved in anti-tumor defense to the tumor site (166).

### 3.4 Anti-LAG3 antibody

LAG3 is a novel immune checkpoint expressed on the surface of CD4^+^ T, CD8^+^ T, NK cells, NKT cells, and regulatory T cells (167, 168). It can be rapidly induced to the cell surface upon T-cell activation. LAG3 has been reported to interact with MHC class II and other ligands such as FGL-1 and galectins (169). LAG3 is highly expressed on CD4^+^ T cells during bacterial infections and can restore their function upon blockade. LAG3 also modulates CD4^+^ T regulatory cells, a subset of T cells that suppress other immune responses (167, 170). Its specific role in cancer, however, remains unclear. The ligand for LAG3, FGL1, accumulates considerably in the prostate tumor tissues and supports rapid tumor growth (171). The expression of the inhibitory receptors, including LAG3, in various treatments is of interest in developing therapies against prostate cancer. A study by Xinyao Zhang et al. identified LAG3 upregulation in the CD4^+^ T cells from PC patients (172).

A DSW Tan et al. study has shown that ieramilimab, an antibody against LAG-3, can inhibit the interaction of LAG-3 with FGL-1. This antibody has been shown to have therapeutic efficacy in treating various cancers, including prostate cancer. The therapeutic use of ieramilimab in patients can increase the production of IFN-γ and the rise in the activity of T cells (173).

### 3.5 Anti-tyrosine-based inhibition motif domain antibody

TIGIT interacts with several PVR/NECTIN family ligands, among which CD155/PVR is considered the most prominent, followed by CD112/NECTIN-2 and CD113/NECTIN-3 (174). The same ligands are targeted by other inhibitory receptors, including the recently described CD96 and CD112R/PVRIG, which dampen NK and T cell activities (175). On the contrary, the costimulatory receptor CD226/DNAM-1 partially shares ligands with TIGIT (176). The expression patterns of TIGIT and CD226 parallel those of CTLA-4 and CD28, with CD226 present on naïve T cells and TIGIT increasing after activation. TIGIT exerts its immune suppressive effects through several mechanisms, including delivering negative signals, competing for ligands that bind with higher affinity to TIGIT, and modifying DCs function (177). TIGIT^+^ T cells are less active than TIGIT^−^ T cells in chronic viral infections, whereas TIGIT^+^ regulatory T cells are more suppressive of effector T-cell activation (178). TIGIT is frequently expressed in human tumors at higher levels in tumor-infiltrating lymphocytes than in those in peripheral blood, suggesting that TIGIT contributes to generating an immune-suppressive tumor environment (179).

Since tumor cells often express TIGIT ligands, targeting TIGIT may be advantageous in cancer therapy. Indeed, studies have demonstrated that TIGIT blockade restrains tumor growth, especially when combined with other ICIs (174, 180). Results from various studies show that prostate cancer TME has high expression of TGIT-related ligands such as CD276, PVR, and NECTIN2 (181). Therefore, they can suppress T-cell responses in this way.

It was identified that the TIGIT monoclonal antibody vibostolimab increases NK cell function against PC by increasing key markers and cytokine production. The TIGIT blockade also increased NK cell signaling pathways and improved T cell attraction to the tumor site. These findings support using TIGIT antibodies and NK cell strategies for PC treatment (182).

Most studies now focus on anti-TIGIT or anti-LAG-3 alone and combination therapies with anti-PD-1 therapies (183, 184). These studies put forth that for therapy against cancer treatment, the combination of anti-TIGIT and anti-LAG-3 may provide a novel approach. A study by Dai et al. showed that ZGGS15 (IgG4 bispecific antibody targeting TIGIT and LAG-3) has potent functional binding and blocking activity against TIGIT and LAG-3. The results showed that ZGGS15 has a high affinity to human LAG-3 and TIGIT, specifically binding to activated CD4^+^ and CD8^+^ T cells with near-equal strength (184). Moreover, ZGGS15 outcompeted LAG-3 and MHC-II, as well as TIGIT and CD155, indicating potential advantages in patients with high levels of FGL1 who tend to have poor outcomes after receiving current treatments. It binds to prostate cancer cell-expressed MHC-II and CD155 and the inhibitory receptors LAG-3 and TIGIT on immune cells. Studies carried out in mouse models reveal that ZGGS15 improves responses by T cells and potentiates the anti-tumor efficacy (184).

## 4 ICIs combined treatments

### 4.1 Dual immune checkpoint inhibitor applications

Studies have shown that, under certain conditions, using a single ICI can lead to compensatory increased expression of other ICIs (185). For this reason, it is recommended to use a combination of two or three ICIs for the treatment of prostate cancer (186). Also, basic immunological studies indicate that immune checkpoint receptors are co-expressed in self-antigen tolerance, chronic infectious disease, and inflammation. In addition to lymphocyte-intrinsic inhibitory receptors, there are two presumed inhibitory ligands from the B7 family, B7-H3 and B7-H4, with no assigned receptor (187). Studies on mouse prostate cancer models have shown that inhibiting a particular ligand or its receptor improves anti-tumor immunity (185).

As mentioned earlier, several receptors, including LAG3, 2B4, BTLA, TIM3, and A2aR, negatively regulate lymphocyte activity and, under appropriate circumstances, anergic lymphocytes (188, 189). Antibody targeting of these receptors enhances anti-tumour immunity in tumor models. Since many tumor cells express more than one inhibitory ligand, several opportunities exist to enhance immune responses by dual or triple checkpoint blocking (190). However, it is essential to know that in some ICI combinations it is possible to overcome implications. For example, in the CheckMate 650 trial, combining anti-CTLA-4 and anti-PD-1 showed an objective rate of response of 25% and 10% in pre- and post-chemotherapy groups, respectively. But patients developed adverse effects in 42%-53% (dose dependent), necessitating dose adjustments (191). While human antibodies targeting some of these inhibitory receptors are being developed, none have yet reached clinical use. Most of these receptors are activated during T cell activation, suggesting they regulate T cell responses when their corresponding ligands are present (**Table 3**).

**Table 3:** Example of immune checkpoints inhibitors application in combination with other treatments for prostate cancer in phase 3 clinical trials.

Study name	Intervention Model, Masking	Estimated Enrollment	Intervention	Date	Status	Sponsor	NTC number	Main findings
A Study of Atezolizumab (Anti-PD-L1 Antibody) in Combination With Enzalutamide in Participants With Metastatic Castration-Resistant Prostrate Cancer (mCRPC) After Failure of an Androgen Synthesis Inhibitor And Failure of, Ineligibility For, or Refusal of a Taxane Regimen (IMbassador250)	Parallel Assignment None (Open Label)	759	1. Atezolizumab 2. Enzalutamide	2024	Completed	Hoffmann-La Roche	NCT03016312	Result in longer progression-free survival in patients
Study of Pembrolizumab (MK-3475) Plus Enzalutamide Versus Placebo Plus Enzalutamide in Participants With Metastatic Castration-resistant Prostate Cancer (mCRPC) (MK-3475-641/​KEYNOTE-641)	Parallel Assignment Masking: Triple	1244	1. Pembrolizumab 2. Enzalutamide	2024	Active, not recruiting	Merck Sharp & Dohme LLC	NCT03834493	Study results have not been submitted
Study of Pembrolizumab (MK-3475) Plus Docetaxel Versus Placebo Plus Docetaxel in Chemotherapy-naïve Metastatic Castration-resistant Prostate Cancer (mCRPC) (MK-3475-921/​KEYNOTE-921)-China Extension	Parallel Assignment Masking: Triple	81	1. Pembrolizumab 2. Docetaxel 3. Prednisone 4. Dexamethasone	2024	Terminated	Merck Sharp & Dohme LLC	NCT04907227	The obtained data did not support study endpoints
Study of Pembrolizumab (MK-3475) Plus Docetaxel Versus Placebo Plus Docetaxel in Chemotherapy-naïve Metastatic Castration-resistant Prostate Cancer (mCRPC) (MK-3475-921/​KEYNOTE-921)	Parallel Assignment Masking: Triple	1030	1. Pembrolizumab 2. Docetaxel 3. Prednisone	2024	Completed	Merck Sharp & Dohme LLC	NCT03834506	
Efficacy and Safety of Pembrolizumab (MK-3475) Plus Enzalutamide Plus Androgen Deprivation Therapy (ADT) Versus Placebo Plus Enzalutamide Plus ADT in Participants With Metastatic Hormone-Sensitive Prostate Cancer (mHSPC) (MK-3475-991/​KEYNOTE-991)-China Extension	Parallel Assignment Masking: Double	186	1. Pembrolizumab 2. Enzalutamide 3. Androgen Deprivation Therapy	2024	Active, not recruiting	Merck Sharp & Dohme LLC	NCT04934722	Study results have not been submitted
Efficacy and Safety of Pembrolizumab (MK-3475) Plus Enzalutamide Plus Androgen Deprivation Therapy (ADT) Versus Placebo Plus Enzalutamide Plus ADT in Participants With Metastatic Hormone-Sensitive Prostate Cancer (mHSPC) (MK-3475-991/​KEYNOTE-991)	Parallel Assignment Masking: Double	1251	1. Pembrolizumab 2. Enzalutamide 3. Androgen Deprivation Therapy	2024	Active, not recruiting	Merck Sharp & Dohme LLC	NCT04191096	Study results have not been submitted
Study of Immunotherapy to Treat Advanced Prostate Cancer	Parallel Assignment Masking: Quadruple	988	Radiotherapy (RT) plus ipilimumab	2016	Completed	Bristol-Myers Squibb	NCT00861614	1. No significant difference in overall survival between ipilimumab and placebo groups 2.Signs of activity with the drug warrant further investigation
Phase 3 Study of Immunotherapy to Treat Advanced Prostate Cancer	Parallel Assignment Masking: Quadruple	837	Ipilimumab	2016	Completed	Bristol-Myers Squibb	NCT01057810	Increased median overall survival
A Study of Nivolumab or Placebo in Combination With Docetaxel in Men With Advanced Castration-resistant Prostate Cancer (CheckMate 7DX)	Parallel Assignment Masking: Quadruple (Double-Blinded)	1030	1. Nivolumab 2. Docetaxel 3. Prednisone	2024	Completed	Bristol-Myers Squibb	NCT04100018	Study results have not been posted
Study of Cabozantinib in Combination With Atezolizumab Versus Second NHT in Subjects With mCRPC (CONTACT-02)	Parallel Assignment None (Open Label)	575	1. Cabozantinib 2. Atezolizumab 3. Abiraterone Acetate	2024	Active, not recruiting	Exelixis	NCT04446117	Study results have not been posted
A Trial of Immunotherapy Strategies in Metastatic Hormone-sensitive Prostate Cancer	Parallel Assignment None (Open Label)	135	1. Ipilimumab 2. Nivolumab 3. Docetaxel 4. Androgen deprivation therapy	2022	Unknown status	Spanish Oncology Genito-Urinary Group	NCT03879122	No result posted

### 4.2 ICIs combined with chemotherapy

Docetaxel is generally given after resistance develops to either abiraterone or enzalutamide in patients with metastatic PC (192). A phase 1b/2 study by Evan Y Yu et al. assessed the efficacy and safety of adding pembrolizumab (anti-PD-1 Ab) to a combination of docetaxel and prednisone (NCT02861573) (193). The measured outcomes of this study are safety, PSA response rate, and ORR. In 104 patients, the confirmed PSA response rate was 34%, and ORR was 23%. This combination regimen demonstrated antitumor activity with manageable safety in chemotherapy-naïve metastatic PC (194). In a phase 1/2 study, the bispecific antibody Vudalimab, which binds to CTLA-4 on one side and PD-1 on the other, was used in combination with docetaxel to treat metastatic prostate cancer (195).

Another study tested combined ICIs in patients with chemotherapy-naïve metastatic PC to the bone. Patients received tremelimumab (anti-CTLA-4 Ab) and durvalumab (anti-PD-1 Ab) every four weeks in combination with chemotherapy. Results showed that 42% experienced grade ≥3 treatment-related adverse events, and 24% had stable disease for over six months. Median overall survival was 28.1 months. The findings suggest that the combination may require additional treatments to address resistance in the bone environment (196).

### 4.3 ICIs combined with radiotherapy

Radiotherapy has been demonstrated to have both immune-enhancing and immunosuppressive effects at the tumor site and systemically (197). In the wake of several promising preclinical studies, several clinical trials were launched, testing the hypothesis that radiotherapy in combination with ICIs would augment anti-tumor immunity. However, many trials have not resulted in impressive gains (198).

Radium-223 dichloride is a treatment primarily directed at bone metastases that improves overall survival in patients with prostate cancer (PC). In one study, 44 patients received a combination of atezolizumab (anti-PD-L1 Ab) with radium-223 in a safety-testing exercise that did not return clinically meaningful benefits, as evidenced by an overall response rate of 6.8% and a median overall survival of 16.3 months (199).

Radiotherapy can cause tumor shrinkage in areas distant from the primary tumor, known as the abscopal effect (200). A study with 28 patients evaluated the combination of ipilimumab (anti-CTLA-4 Ab) and radiotherapy, resulting in one complete response and several stable disease cases (201). A larger trial compared ipilimumab to placebo in 799 patients, showing similar median survival rates. However, longer follow-ups indicated better survival rates with ipilimumab at 2 and 5 years. Adverse effects included severe gastrointestinal issues, and some deaths were due to the treatment (202).

In another study, ^90^Y-NM600 was used as a targeted radionuclide therapy (TRT) mediator in combination with an anti-PD-1 antibody to treat prostate cancer in an animal model. Surprisingly, that study showed that this combination was ineffective in treating PC because the anti-PD-1 antibody activated and expanded Treg cells (203). Indeed, the triple combination of anti-CTLA-4/anti-PD-1/radiotherapy showed the best survival and tumor growth delay compared to dual and single therapies. Thus, the preclinical model suggests that this combination is effective and must be pursued in a clinical setting for PC (204).

Therefore, although this combination presents challenges, such as the abscopal effect, and can lead to “off-target” anti-tumor effects, it is used in many clinical studies to treat PC. The clinical trial studies using this combination are NCT04446117, NCT04946370, NCT04931979, and NCT03543189. However, the results of these studies are not yet available.

### 4.4 Other combinations

#### 4.4.1 Androgen deprivation therapy

One of the main treatments for advanced prostate cancer is ADT, which causes immune alterations in the tumor microenvironment. Thymic regeneration, reduced tolerance, and an improved adaptive immune response can facilitate immune cell infiltration after ADT (205). However, these positive effects are transient and may be counterbalanced by an increased immunosuppressive cell compartment. Thus, this provides a rationale for combining it with ADT. The PRIME-CUT Phase II trial is investigating the combination of ADT and cemiplimab (anti-PD-1 Ab) treatment in men with metastatic castration-sensitive prostate cancer (206, 207). The results of this study led to the introduction of a new concept of “Highly Active Anti-Tumor Therapy” HAATT, including vigorous early treatment to eradicate resistant tumor cells, enhance the function of CD8^+^ T immune cells, reduce regulatory T cells, and interfere with growth-promoting substances in the tumor environment (206).

#### 4.4.2 Electroporation

Some studies have also used more creative methods to develop an ICI-based therapy. Studies have shown that focused electroporation at the tumor site can damage tumor tissue and kill tumor cells (207). These killed tumor cells release antigens that can activate immune system cells. For this reason, in a study by BJ Burbach et al., the ICI beta electroporation was used to treat PC. For this purpose, after tumor induction in mice by transplantation of TRAMP-C2 cells and treatment by electroporation, a stock solution containing three types of ICI (antibodies against PD-1, PD-L1, and CTLA-4) was used. The results of this study showed that this combination can lead to an increase in the prostate tumor-specific CD8^+^ T cell population (208).

#### 4.4.3 STAT3

The STAT3 signaling pathway contributes to the development of immunosuppressive cells and represses DCs functions within tumors to support immune evasion of cancer (209). It controls immunosuppressive factors of tumor cells and is an attractive drug target, which may enhance the effects of immune checkpoint inhibitors (210).

Therefore, in the study by Kristina Witt et al., a combination of an antibody against CTLA-4 and an inhibitor of the STAT3 signaling pathway (GPB730) was used. The results of this study show that after combination treatment in the PC mouse model, the population of regulatory T cells within the tumor is significantly reduced compared to single treatments. Considering the pathological role of regulatory T cells in tumor progression, a reduction in the population of these cells leads to tumor improvement and a decrease in size (211). In another study, CFF-1 was also used as a treatment for PC. This treatment reduced PSA levels and improved life quality for patients with advanced prostate cancer. In a mouse model, PD-1/PD-L1 increased during tumor growth, reducing CD3^+^ T cell subsets. CFF-1 decreased PD-L1 expression dose-dependently, inhibited tumor growth, and helped recover CD3^+^ T lymphocytes (212). It also extended survival and reduced lung metastasis, especially when combined with docetaxel. CFF-1 can serve as an ICI and STAT3 inhibitor and shows promise as a treatment for prostate cancer by enhancing immune response (212).

#### 4.4.4 Vaccine

Tumor vaccines typically require a booster treatment to improve their efficacy. For this reason, in many cases, adjuvants are used to increase immune responses. However, it seems that using ICI can lead to improved vaccine efficacy. For this reason, in the phase 2 study by G McNeel et al., the MVI-816 vaccine (antigen prostatic acid phosphatase (PAP)) was used in combination with pembrolizumab for the treatment of PC in patients (NCT02499835) (213). The results of this study show that the use of this combination therapy can lead to the expansion of Th1 cell responses, an increase in the response of specific CD8^+^ T cells, and an increase in the production of IFN-γ and granzyme B by these cells (213).

#### 4.4.5 Cryotherapy

A small pilot study also utilized cryotherapy in combination with pembrolizumab (an anti-PD-1 Antibody). The median progression-free survival was 14 months, with no serious adverse effects (214).

## 5 Challenges, limitations, and future perspectives

ICI therapy has transformed the treatment landscape over the last decade and continues to evolve. Appreciating organ-specific toxicities will remain critical for the practicing oncologist and other specialists who contribute to patient care (215). Endocrine toxicities represent some of the most common adverse effects, and therefore, management by the endocrinologist becomes paramount (216). This awareness will further grow with the increased use of ICI regimens. Some of the research challenges concerning ICI-induced endocrine disorders are finding predictors and risk factors of these effects, such as pituitary dysfunction, which will help select cancer treatment and enhance monitoring and prevention strategies (217). The other challenge is investigating the mechanisms behind the endocrine syndromes associated with ICIs. Investigation into thyroid inflammation might provide some insights and further opportunities for study (218). Finally, investigating the relationship between thyroid dysfunction and improved survival in cancer is also warranted (216).

Also, the long-term effects of ICIs are becoming increasingly important (219). Whereas most studies have focused on short-term toxicities, new evidence suggests that long-term effects may be more common than previously thought. Chronic toxicities, in general, affect all organ systems, including endocrine and rheumatological issues (219). Given the potential for long-term survival, the development of fatal toxicities is 0.4–1. 2% of patients is not trivial. Moreover, ICIs touch many aspects of immune function, and their long-term impact on cancer survivors remains unknown mainly (220).

Developing approaches to predict the risk of immune-related adverse events (irAEs) is critical for optimizing ICI therapies or switching patients to less dangerous treatments (221). Some studies have demonstrated that cytokines may amplify the development of irAEs, and therapies targeting such cytokines have been used in clinical practice to manage severe irAEs (222). Most irAEs are mild, but early detection and treatment are crucial. Nurses are essential in identifying irAEs and educating patients on symptoms (223). In addition to monitoring, it is also essential to control these events in patients through pre-treatment examinations and post-treatment measures. The Common Terminology Criteria for Adverse Events (PRO-CTCAE) version for patient-reported outcomes effectively lowers hospitalization rates, improves quality of life, and facilitates survival (224). Limited data regarding its use in monitoring irAEs are available. In a trial with 16 PC patients, irAE-related items from NCI’s PRO-CTCAE were used alongside CTCAE (224). Symptoms like fatigue and rash were often reported, with greater irAE severity noted in PRO-CTCAE. Further study is needed on the role of monitoring programs in irAE management.

However, it is tough to establish a universal strategy for preventing irAEs since patients may respond differently. The gut microbiome and its metabolites have shown good interactions with ICI therapy and reduced side effects associated with such drugs. Both preclinical studies and clinical data warrant that the gut microbiome may help manage the development of irAEs (225).

The gut microbiota differed in mice lacking the PDCD-1 receptor, with a marked decrease in *Lactobacillales* (226). A model highlighted that Lactobacillus salivarius is a key species predictive of irAEs. Other anti-inflammatory probiotics include *Bifidobacterium* and *Lactobacillus* (227). Two species of *Parabacteroides* and two of *Ruminococcus* were identified as playing a key role in predicting the onset of irAEs (228). Lower menaquinone biosynthesis has been shown in patients with irAE compared to non-irAE (225). This may indicate the potential for either gut microbiome modulation or metabolites such as menaquinone to prevent irAE. Further research is needed to establish if low levels of menaquinone contribute to developing irAEs. It is still under debate whether treating irAEs diminishes the effectiveness of the immunotherapy itself. Biomarker identification regarding irAE could allow for better therapeutic efficacy while limiting adverse events. A better understanding of gut microbiome interactions and their roles in irAE will require more rigorous experimental designs and further validation in future studies.

Another point that is raised in the treatment of different cancers by ICIs is the variation in the efficacy of this type of treatment (229). Several factors can affect this efficacy and can include the ICIs surface expression level (like PDL1), the level of tumor mutational burdens, as well as the degree of penetration of the antibody into the tumor tissue (230). These factors lead to better responses in head and neck, lung, and gastro-esophageal junction cancers with elevated mutation burden (230). Main metastatic cancers also responded well because they typically contain high mutational burdens. This seems to be one of the reasons why the success rate of PC treatment using ICIs differs from other cancers (231). Given the variations seen in ICI treatment across different cancers, the Yoo and colleagues research group developed an AI-based algorithm to predict ICIs-based treatment outcomes (232). Predicting whether cancer patients will benefit from ICI without advanced testing is important. The group developed SCORPIO, a machine learning system that uses routine blood tests and patient data from 9,745 patients treated with ICI across 21 cancer types. SCORPIO was trained on 1,628 patients from Memorial Sloan Kettering Cancer Center (232). The method outperformed tumor mutational burden in predicting overall survival and clinical benefit in two sets of trials.

## 6 Conclusion

Given the impact of PC on patients’ quality of life and its mortality rate, there is a need for an effective treatment to prevent tumor progression. Current treatments are insufficient. Since the immune cell response can halt tumor growth, immunotherapy measures can assist in treating this disease. Immunotherapy is typically administered using cells or antibodies. With the advent of ICIs and their integration into tumor treatment, they have been extensively utilized in managing PC. Although the outcomes have been promising, some challenges include the induction of treatment resistance, the emergence of endorphin responses, and their toxicity in various tissues. Therefore, researchers have sought to combine different therapies with ICIs. To date, treatment modalities such as chemotherapy, radiotherapy, cryotherapy, PARP inhibitors, vaccines, ADTs, and several other therapies have been combined with ICIs to inhibit tumor growth in preclinical and clinical trials. Examining biomarkers such as microbiome composition, tumor gene mutation burden (TMB), microsatellite instability (MSI), and DNA-repair gene mutations that result in reduced DNA mismatch repair (MMR), as well as target protein expression in PC patients and tumor tissue, can significantly assist in selecting appropriate treatment based on ICIs. These biomarkers show us the potential for tumor cell transformation, and in general, it seems that MMR-deficient and MSI-high tumors are better responders to ICI treatment (233). Combination therapies prevent tumors from escaping treatment by targeting different mechanisms. However, research in this area is ongoing, and further studies are required to confirm an effective therapeutic combination with minimal side effects.
